# The Seeds of Death

**Published:** 1870-10

**Authors:** 


### THE SEEDS OF DEATH.

"The moment when our lives begin,

We all begin to die."

Every man has within himself the seeds of decay, decrepitude and death, and so with all nations of past history. Greece and Rome perished from the face of the earth because they had within themselves the elements of destruction, without adequate antagonizing influences. We, in our pride, may think that a different destiny awaits the United States, and we pride ourselves in our educational institutions, our wide domain, and our internal resources, of soil, and ores, and sea coasts. Education is a sword which cuts two ways ; it simply gives a man greater capabilities for good or evil. Education and culture did not save Greece and Rome, nor did wealth, nor power, nor military prestige. Wealth brought extravagance, and indulgence, and effeminacy. There is nothing that can save or will save the United States from dismemberment, and anarchy, and destructive civil wars, from preying on one another, but the elements of the Christian religion ; and these elements, like good seed, must be sown in the hearts of children, beginning with infancy. But there is something more to be done. We must eliminate the seeds of death, just as the skilled physician is careful to remove the causes of disease, and sometimes must cut out the malignant tumor. We know very well that there are poisons which enter the system at a point not larger than a pin's head, and a single drop of the virus works death. The moral poisons which now threaten this country are :

First, Laxity of Sabbath observance.

Second, Laxity of view as to the marital relation.

Third, Laxity of opinion as to Bible authority.

Fourth, Laxity of family religious culture.

That is a seed of national death whose fruitage as to the Sabbath day is the Sunday excursion ; the sacred concert ; the walk in the fields ; the drive to the park ; the idle walk ; the open library and free reading-room.

That is a seed of death whose fruitage is the practice of divorce for less cause than the Bible warrant of adultery.

That is a seed of death whose fruitage, as to the Bible, is to regard it less than a divine revelation, imperative and absolute, the ultimate authority in all human duty.

That is a seed of death whose fruitage, as to the children of the Church, tends to commit the religious teaching of the children of Church members to any others than father and mother ; and to keep the Bible and Prayer Book out of the school-house.

There are clergymen whom we know are sowing these four kinds of seeds of death ; some one, some another, some all ; men of might some of them are, men of renown and no might, as are others. To the pulpit and the religious newspapers we must look. Both must have more power—that is, more concentrated talent in both, and to this end the wealth of the Church ought to flow; to pay the minister well while he does work, and to pension him handsomely when he can work no longer. As to the religious press, half the religious newspapers so called should be bought up or allowed to die for want of support, and the whole patronage of the church should be concentrated on a few organs, in such a way that the highest price could be paid for contributions—thus commanding the highest talent of the clergy. An immense circulation would follow, and with that an advertising patronage could be secured which would make the press of the Church a source of income, to be expended in cheapening religious instruction and intelligence.

